# Neuropsychological Test Pattern Associated With a High Percentage of Sleep Apnea Diagnosis

**DOI:** 10.1002/brb3.71097

**Published:** 2025-12-22

**Authors:** Robert J. Przybelski, Anna G. Przybelski, Meredith E. Rumble, David T. Plante, Alissa M. Irwin, Jana E. Jones

**Affiliations:** ^1^ Department of Medicine University of Wisconsin School of Medicine and Public Health Madison Wisconsin USA; ^2^ Center for Psychedelic and Consciousness Research Johns Hopkins University Baltimore Maryland USA; ^3^ Department of Psychiatry University of Wisconsin School of Medicine and Public Health Madison Wisconsin USA; ^4^ Jesse Brown Department of Veterans Affairs Medical Center Chicago Illinois USA; ^5^ Department of Neurology University of Wisconsin School of Medicine and Public Health Madison Wisconsin USA

**Keywords:** cognition, delayed memory, geriatrics, immediate memory, memory, OSA

## Abstract

**Introduction:**

Obstructive sleep apnea (OSA), common in geriatric patients, has been associated with neurocognitive memory disorders such as Alzheimer's disease and other related dementias. An unusual pattern of memory testing has been found to be associated with OSA in a retrospective study (Dexter and Ebert, 2019). This atypical pattern is reported when immediate memory performance is lower than delayed memory performance on the repeatable battery for the assessment of neurological status (RBANS). The current study examines this cognitive testing results pattern coupled with snoring for predicting OSA in geriatric memory patients presenting to a university‐based memory clinic.

**Methods:**

A convenience sample of patients presenting to the University of Wisconsin Hospitals and Clinics geriatric memory clinic from 2016‐2020 for cognitive assessments comprised the study population. Patients completed the mini‐mental state examination (MMSE) and obtained a score of 25 out of 30 points or greater and the patients were given the RBANS. Patients were referred for a sleep evaluation if they snored and their immediate memory index score was one or more points lower than their delayed memory index score on the RBANS. No other test score requirements were utilized to trigger the referral.

**Results:**

Of the 251 patients (138 men; 113 women) referred based only on these two criteria to the associated sleep clinic, 158 (80%) were found to have OSA.

**Conclusion:**

The prevalence of positive sleep studies suggests that memory clinic patients who snore and present this unusual pattern of results on the RBANS should be referred for a sleep evaluation for possible OSA.

## Graphical abstract

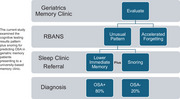

## 1 Introduction

OSA is common in geriatric patients and has been associated with Alzheimer's disease and related dementias. (Ferini‐Strambi et al. 2021) OSA is characterized by upper airway obstruction causing intermittent hypoxia, often disrupting sleep and causing over‐compensatory responses of the autonomic nervous system. (Ferini‐Strambi et al. 2021; Dempsey et al. 2010) This has been reported to lead to inflammatory reactions, oxidative stress, and impaired neurovascular coupling. (Lavie 2003) The hippocampus, playing a pivotal role in memory, has been reported to be especially vulnerable to these phenomena. (Javaheri et al. 2015)

Memory dysfunction in OSA has been characterized by deficits in immediate memory and delayed memory. (Sforza and Roche 2012) Immediate memory deficits in OSA patients are thought to be due to impaired attention, encoding, and initial storage of information, disrupted by hypoxia and sleep interruption. (Canessa et al. 2011; Zhou et al. 2016) Neuropsychological assessments such as the RBANS show that OSA patients perform significantly worse on immediate recall tasks for both verbal and visual stimuli. (Canessa et al. 2011; Zhou et al. 2016) This pattern of poor performance is also seen in delayed memory, where subtests of the RBANS including list recall, story recall, and figure recall are significantly poorer in OSA patients compared to healthy controls. (Canessa et al. 2011; Zhou et al. 2016; Ferini‐Strambi et al. 2003) This impaired performance is thought to indicate delayed memory impairments across verbal and visuospatial domains. (Canessa et al. 2011; Morrell et al. 2003)

Dexter and Ebert (2019) reported a unique pattern of results on the RBANS and proposed an association with obstructive sleep apnea. In a clinical setting where 191 patients completed the RBANS, 81 (42%) displayed immediate memory scores lower than delayed memory scores. Fifty‐four of those patients had been or were subsequently tested for OSA, and 35 (65%) were found to be positive. The authors reported that this pattern was not typically seen in Alzheimer's, Lewy body, front temporal, cerebrovascular, or other neurological diseases typically causing cognitive impairments. (Dexter and Ebert 2019)

This reported pattern may be important for diagnosing geriatric patients with obstructive sleep apnea. It can be difficult to diagnose OSA in older populations as patients are less likely to report classic symptoms such as loud snoring or observed apneas. (Ancoli‐Israel et al. 1991) Further, older adults may have a lower BMI or smaller neck circumference, negating questions on screening questionnaires such as STOP‐Bang that would lead to referral for a sleep study. (Koo et al. 2013; Chung et al. 2008) Geriatric patients are more likely to report daytime sleepiness, fatigue, depression, or cognitive impairment symptoms, which are more commonly attributed to other medical conditions. (Koo et al. 2013; Gooneratne et al. 2011)

The current study examined the relationship between sleep study outcomes of geriatric memory clinic patients referred for sleep studies after demonstrating this unusual pattern on the RBANS, as well as having a complaint of snoring.

## 2 Methods

A convenience sample of patients presenting to the University of Wisconsin Hospitals and Clinics geriatric memory clinic from 2016‐2020 for cognitive assessments comprised the study population. If the screening evaluation with the Mini‐Mental State Examination (MMSE) produced the score of 25 out of 30 points or greater, the patients were given the RBANS. (Randolph 2012) All study procedures were reviewed and approved by the University of Wisconsin‐Madison Institutional Review Board and determined to qualify for exempt status, as it involved only record review with minimal risk.

The RBANS is a commonly used brief neurocognitive battery measuring attention, language, visual spatial/constructional abilities, and immediate and delayed memory. The test is comprised of 12 subsets: List learning, story memory, digit span, coding, figure copy, line orientation, picture naming, semantic fluency, list recall, story recall, and figure recall. These 12 subtests are grouped into five scaled Index Scores and a Total Score. Each Index Score is expressed as a scaled score with a mean of 100 and a standard deviation of 15. The usual RBANS pattern seen with Alzheimer's disease and related dementias has the immediate memory score greater than the delayed score, consistent with accelerated forgetting. (Randolph 2012)

Patients were referred for a sleep evaluation if they snored and their immediate memory index score was one or more points lower than their delayed memory index score on the RBANS. No other test score requirements were utilized to trigger the referral. Neither the STOP‐Bang, Epworth Sleepiness Scale, nor any other screening tool for OSA was used to make the referral, and none of the patients had a prior diagnosis of OSA. It should be noted that Medicare requires the STOP‐Bang cutoff score to be three or greater in order to complete a sleep evaluation. Males receive one point for gender and females do not.

The evaluation for OSA involved either the Home Sleep Apnea Testing (HSAT) or polysomnography (PSG), as determined following the standard clinical triage criteria of the Wisconsin sleep laboratory and clinic associated with UW Health at the University of Wisconsin. HSAT was performed using Alice PDx devices which utilized pulse oximetry, oronasal thermistor, nasal pressure transducer, and thoracic/abdominal effort belts. In laboratory PSG and sleep stage scoring were performed following American Academy of Sleep Medicine (AASM) parameters. Board certified sleep medicine physicians interpreted all sleep studies, and graded the severity of OSA as mild, moderate, or severe. For this study, hypopneas were scored using Center for Medicare and Medicaid (CMS) criteria requiring 4% or more oxygen desaturation. Sleep referrals, completions, OSA diagnosis of severity, age, and sex were obtained retrospectively from the medical record. A chi‐square analysis was conducted to determine if rates of OSA were different between males and females.

## 3 Results

There were 251 patients who were referred for a sleep study with an average age of 75.5 (*SD* = 5.64) years. One hundred fifty‐eight patients received sleep studies as a result of those referrals (93 males and 65 females). As noted in Table 1, of 158 patients studied, 127 (80%) were found to have OSA with a significant difference in OSA diagnoses between males and females (X*^2^* (1, N = 158) = 1.7, p < 0.05). The average age of those with sleep studies was 75.33 (*SD* = 5.77). The number of males with moderate or severe disease was 47 (59%) was also significantly greater than females (X*^2^* (1, N = 145) = 4.5, p = 0.03). Of note, 10% fewer women than men obtained the sleep study even though they were referred.

**TABLE 1:** Patients referred for sleep evaluation.

—	Patients referred	Patients studied	OSA diagnosed	Moderate/severe OSA
Male	138	93 (67%)	80 (86%)*	47 (59%)*
Female	113	65 (57%)	47 (72%)	25 (53%)
Total	251	158 (63%)	127 (80%)	72 (57%)

*Note*: (*p < 0.05).

## 4 Discussion

This retroprospective record review study suggests that the unusual RBANS pattern of the immediate memory score less than the delayed memory may be a significant indicator for OSA, along with the addition of the symptom of snoring in geriatric patients referred from a memory assessment clinic. These were patients that had not previously been diagnosed with OSA. These referrals were based on the unusual immediate and delayed memory test pattern plus the presence of snoring, these patients may have never been referred for a sleep study and potentially never treated for this serious condition. And, as treatment for OSA has been shown to improve memory performance as well as reduce cardiovascular and cerebrovascular risk, the patients obtaining treatment will likely function better cognitively than if they had not been referred for the sleep study. (Dexter and Ebert 2019; Canessa et al. 2011; Zhou et al. 2016)

One of the hallmark symptoms of OSA is brief arousals due to repetitive lapses in breathing pattern secondary to air flow cessation. Impairments and attention/executive functioning have been associated with sleep fragmentation and hypoxia/hypercarbia. (Dempsey et al. 2010; Sforza and Roche 2012) It is widely known that these cognitive skills are essential for successful encoding of information, as indicated by the immediate memory score in the RBANS. (Ferini‐Strambi et al. 2003; Randolph 2012) As such, it is reasonable to believe that the pattern of impairments seen on the RBANS test of worse immediate memory than delayed memory could identify OSA as a discrete condition in the memory patients being tested. (Dexter and Ebert 2019)

It was noted that 10% fewer women received the requested sleep study than men who were referred. One possible explanation for this discrepancy is that the criteria used to support CMS and insurance reimbursement for the sleep study could not be met for women as easily as for men. More specifically, the STOP‐Bang questionnaire, which provides an extra point of significance to the male sex, is the most commonly used criterion for prior authorization for a sleep study. (Sforza and Roche 2012; Chung et al., 2008) Thus, compared to men, one out of 10 women will go untested and untreated if only the STOP‐Bang continues to be used to qualify them for a sleep evaluation. (Koo et al. 2013; Chung et al., 2008)

A significant limitation of this study is that only patients who had mild memory problems, as determined by the score of 25 or greater on the MMSE, were administered the RBANS. Therefore, patients with more significant memory problems were not included in this chart review. Patients with moderate to severe memory problems might not show the same unusual RBANS pattern particularly since their delayed memory is likely to be significantly lower than what was seen in this patient sample. (Dexter and Ebert 2019)

In terms of the sleep evaluation, the use of a clinical sample increases generalizability of the study findings, but this also caused sleep disordered breathing assessments to be conducted according to clinical care pathways that used both home and in‐laboratory sleep testing, which may have impacted the final sleep diagnoses. Additionally, as a retrospective chart review study, there is not a direct comparison group against which the frequency of OSA can be compared.

Future research that clarifies the test characteristics of neuropsychological testing and symptom pattern for sleep disordered breathing is indicated. Follow‐up of the patients referred for evaluation, including retesting with the RBANS, could determine if the pattern persists, and if in fact patients appear to improve on their test performance if they receive treatment for their previously undiagnosed OSA. Additionally, it will be important to examine other metabolic parameters like body mass index, diabetes, and hypertension.

## 5 Conclusion

Medical providers should maintain a high degree of suspicion for OSA when immediate memory is more impaired than delayed memory on the RBANS, especially in geriatric patients who snore. These patients should be referred for a sleep evaluation.

## Author Contributions

**RJ Przybelski**: conceptualization, data curtion, formal analysis, investigation, methodology, project administration, resources, supervision, validation, visualization, writing, review and editing. **AG Przyelski**: data curation investigation, project administration, methodology, resources, writing, review and editing. **M Rumble**: investigation, methodology, writing, review and editing. **DT Plante**: investigation, methodology, resources, supervision, validation, visualization, writing, review and editing. **AM Irwin**: data curation investigation, methodology, resources, writing, review and editing. **JE Jones**: conceptualization, data curtion, formal analysis, investigation, methodology, resources project administration, supervision, validation, visualization, writing, review and editing.

## Funding

The authors have nothing to report.

## Conflicts of Interest

The authors declare no conflicts of interest.

## Data Availability

The data that support the findings of this study are available from the corresponding author upon reasonable request.
